# Ocean Science Diplomacy can Be a Game Changer to Promote the Access to Marine Technology in Latin America and the Caribbean

**DOI:** 10.3389/frma.2021.637127

**Published:** 2021-04-12

**Authors:** Andrei Polejack, Luciana Fernandes Coelho

**Affiliations:** ^1^ WMU-Sasakawa Global Ocean Institute, World Maritime University, Malmö, Sweden; ^2^ Ministério da Ciência, Tecnologia e Inovações, Brasília, Brazil; ^3^ Research Group Natural Resources, Law, and Sustainable Development, Brazilian Institute for the Law of the Sea, Salvador, Brazil

**Keywords:** science diplomacy, access to technology, Latin America, caribbean, UN decade of ocean science

## Abstract

Ocean science is central in providing evidence for the implementation of the United Nations Law of the Sea Convention. The Convention’s provisions on transfer of marine technology to developing countries aim at strengthening scientific capabilities to promote equitable opportunities for these countries to exercise rights and obligations in managing the marine environment. Decades after the adoption of the Convention, these provisions are under implemented, despite the efforts of international organizations, such as IOC-UNESCO. Latin America and the Caribbean struggle to conduct marine scientific research and seize the opportunities of blue economy due to the limited access to state-of-the-art technology. Ocean science communities in these countries are subject to constraints not foreseeing in international treaties, such as unstable exchange rates, taxation, fees for transportation, costs of maintenance and calibration of technology, challenges to comply with technical standards, and intellectual property rights. Action is needed to overcome these challenges by promoting a closer tie between science and diplomacy. We discuss that this interplay between science and international relations, as we frame science diplomacy, can inform on how to progress in allowing countries in this region to develop relevant research and implement the Convention. We provide concrete examples of this transfer of marine technology and ways forward, in particular in the context of the UN Decade of Ocean Science for Sustainable Development (2021–2030).

## Introduction

For the past decades, as the same time as scientific discoveries allowed us to acknowledge the critical importance of the ocean to our livelihood, it was also significant to demonstrate the serious consequences of anthropogenic impacts on the marine environment threatening this life-supporting system (Rockström et al., 2009). It is a humanitarian solicitude to preserve and sustainably use the ocean, conserving its essential ecosystem services for generations to come (Griggs et al., 2013). However, science and technology have not served all countries equally (Harden-Davies and Snelgrove, 2020; Ocampo and Vos, 2008, pp. 34–36). As the UN Decade of Ocean Science for Sustainable Development makes its debut, this paper seeks to assist it by discussing current limitations hampering countries in Latin America and the Caribbean from accessing and using marine technologies to develop the science needed to inform decisions and international negotiation processes in an equitable basis.

Science has been responsible for both acknowledging the critical importance of the ocean as well as identifying its multiple stressors and delicate ecological limits (Nash et al., 2017). With the increasing significance of environmental and ocean related discussions in international fora, scientists are called to provide evidence on life-threatening issues, such as natural and human induced hazards or food security and pollution. More recently, science has been pushed in the ocean international arena to assume a more relevant social role rather than just unveiling the unknowns (Wisz et al., 2020). Scientists are requested to provide empirical inputs to global decision-making processes, with the potential to build international partnerships to overcome these collective humanitarian challenges (Fedoroff, 2009). Ocean scientists are also being urged to deliver social goods and foster capacity development and transfer of marine technology (IOC-UNESCO, 2020b)^1^. Nevertheless, ocean knowledge production depends upon the access and application of available marine technologies. These include not just research vessels, underwater vehicles and oceanic instruments, but all sort of expertise and knowledge-based materials, including databases and information, as formatted by the Intergovernmental Oceanographic Commission (IOC) of UNESCO (IOC-UNESCO, 2005). Therefore, accessing marine technologies is critical to develop ocean research that can ultimately provide evidence to decision-making.

Developing countries struggle to develop or access marine technologies in spite of some attempts to address this issue (Alexander et al., 2020). Vast ocean areas are still unmapped and unknown to humanity, in particular the Southern parts of the Atlantic and of the Pacific, mostly due to the lack of access to marine technologies and incipient human capacities of countries in these regions (Inniss et al., 2017; IOC-UNESCO, 2017). The asymmetrical distribution of scientific knowledge and technologies not only impinge discoveries, but also reduce possibilities of developing countries to table their needs in international negotiations on ocean affairs based in sound evidence. As one of the major historical battlefields between developing and developed countries, the United Nations Convention on the Law of the Sea (LOSC) enshrines provisions to promote international cooperation on marine scientific research (MSR) and the transfer of marine technology (TMT)^2^ (Anand, 1982; Soons, 1982; Nordquist et al., 1990; Gorina-Ysern, 2004). However, these provisions are among the less implemented in the LOSC (Long, 2007; Long and Chaves, 2015; Salpin et al., 2018).

Enforcing the LOSC rules on MSR and TMT in an equitable manner has been in the forefront of the international agenda for developing countries, as for instance in the current negotiations of a legally binding implementing agreement to regulate the conservation and sustainable use of marine biodiversity beyond national jurisdiction (BBNJ agreement) (Long and Chaves, 2015; Harden-Davies, 2018). The UN Decade of Ocean Science also lies within this background, focused on balancing countries’ capabilities to promote sound science for social and environmental benefit. Nonetheless, it is uncertain how the geopolitical interactions between the actors negotiating these processes will occur, as well as which roles will be played by scientific evidence.

The Decade is a diplomatic movement to foster marine research in search of fulfilling the targets established under the Sustainable Development Goal 14, Life below Water (SDG14), in which ocean science is pivotal (Visbeck, 2018). As a coordination effort to this end, the Decade will need to deal with the transfer of marine technology to the Global South, without which ocean science cannot progress globally as requested. The Decade’s ambition to involve other ways of knowing in science making, plus improving this knowledge uptake in society’s decision making, will need to involve social scientists further (Ryabinin et al., 2019). Social sciences are called to the front to ask the correct questions and bridge all ways of knowing (Claudet et al., 2019). In this context, science diplomacy will be pivotal for the Decade’s success.

International Relations scholarship has overseen the role of science and technology in theorizing the relations of power and influence between countries (Mayer et al., 2014). Globalization, for instance, has been mostly researched in economical contexts, whereas science has been described as an influential soft form of power, attracting partner countries to one’s interests and values, rather than using force and coercion (Nye, 2017). Science diplomacy is a recent field of academic research that investigates exactly the relationship between science and international relations, opening a new horizon for scholarship in International Relations (The Royal Society, 2010; Gluckman et al., 2018; Rungius et al., 2018). Although its definition is still disputed [a good debate can be found in Flink (2020) and in Ruffini (2020b)], for the purpose of this piece, science diplomacy is framed as a practice by which international relations support and are supported by scientific research, evidencing sometimes conflicting national, regional, and global interests. The current debate around the topic has provided insightful perspectives to think about fostering the access to marine technology for developing countries (Griset, 2020).

This paper assesses how science diplomacy can be a significant tool for Latin America and Caribbean States to overcome challenges in negotiations related to accessing marine technologies and capacity building at the international level, ultimately enhancing the regions scientific capacities. Profiting from the opportunity presented by the implementation of the UN Decade of Ocean Science for Sustainable Development (2021–2030), we propose recommendations that could leverage the implementation of the legal rights and obligations on transfer of marine technologies reducing global inequalities in the access and use of marine technologies.

## Methods

We conducted a legal analysis of the provisions adopted in the LOSC regarding the promotion of MSR and TMT, focusing on the rules with especial provisions for developing countries. Additionally, official documents aiming at implementing such provisions were analyzed, in particular those from the Intergovernmental Oceanographic Commission from UNESCO (Gonçalves, 1984; Harden-Davies and Snelgrove, 2020). Some of the perspectives and examples provided were drawn from the authors’ experience in managing scientific programs in the region and through the collection of views from researchers in the field over time. We acknowledge the importance of analyzing how social, cultural and political relations can add layers of complexity in the discussion of implementing the transfer of marine technology obligations, however, this has not been the focus of this paper.

### Reasons Why Marine Technology Transfer Is Critical in Latin America and the Caribbean

Globalization is usually themed after economic relations but became a facilitator movement of international scientific cooperation, in particular in issues of global concern, such as ocean health (Held et al., 1999; Carter, 2008). With a more engaged global scientific community, the knowledge produced could reflect a form of scientific consensus that could inform diplomacy. However, the uneven participation of researchers from Latin America and the Caribbean in global ocean assessments show that this consensus might be reflecting views from a narrow group of scientists, lacking inclusivity (IOC-UNESCO, 2020a; Tessnow-von Wysocki and Vadrot, 2020). Thus, globalization has provided good opportunities for the evolution of Science but has still much to progress in terms of accommodating knowledge from other communities, in particular researchers from the Global South (Biermann and Möller, 2019; Kraemer-Mbula et al., 2020).

Researchers from developed countries often access funding and infrastructure to conduct research in Latin America and the Caribbean waters. As principal investigators of such research projects, these researchers usually apply only a small portion of the funding in the foreign field, leaving local contributors with limited access to research equipment. This has been evident in the current Covid-19 pandemic, with Northern scientists regretting having lost their field work access due to travel bans, thus jeopardizing entire research projects (de Vos, 2020). What should be regretted is that those research projects did not provide a well-equipped and trained personnel on the ground. If done so, research would have been preserved, so as capacity development and access to technology provided, a win-win situation.

Ocean scientists in Latin America and the Caribbean struggle in many ways to develop world-class marine research. First, research budget is limited and allocated in local currency, subject to high fluctuating exchange rates. This conversion is necessary to import equipment and other research inputs from foreign companies, usually from developed countries. Research proposals’ budget are challenged in predicting this currency fluctuation as well as adding the high costs related to taxation and transportation. As a result, research inputs and equipment can become prohibitive. Managing these discrepancies becomes a fundamental part of doing ocean science in the Global South.

Second, once an equipment is imported, it needs to be calibrated and maintained by certified services so results can be compared, and data defined as accurate. In general, these certified services are only provided by the same companies that manufacture the devices. The contracting party is usually hold accountable to cover the costs of the technician’s travel and accommodation, plus the service itself. Establishing local or regional offices in the region would provide not only a solution, but also foster jobs and boost small enterprises and start-ups. Ocean technology companies claim that the market share in Latin America and the Caribbean is insufficient for opening branches in the region. Indeed, limited funding results in less acquisition of equipment, making the market share low for those companies. Countries could develop certified laboratories to provide maintenance and calibration. Brazil, for example, has this capacity established in universities. Those laboratories are however unable to be certified due to the high international standards for accreditation, costly to comply with. Without this certification, one can just loose the equipment’s warranty or have the data being trashed out for the lack of quality assurance.

Lastly, the global ocean scientific community moves steadily in determining essential ocean variables, i.e., a minimum requirement of observations to monitor the state of the ocean environment and predict trends which are useful to inform society and policy makers (Lindstrom et al., 2012). It has been acknowledged that complying with such standards will be challenging to the developing world, in particular because of the fragmented ocean international governance framework and the lack of coordination and security in funding schemes (Bax et al., 2018). Capacity development and transfer of marine technology are critical to instrumentalize a coordinated set of data that will allow better forecast and modeling of the marine environment (Miloslavich et al., 2018). Despite some endeavors in the Pacific and Southern Asia (Bax et al., 2018), the overall scenario in ocean observations is still detrimental (Tanhua et al., 2019).

All in all, ocean scientists in the South have limited research budget in local currency with highly fluctuating exchange rates. Much of this budget is then spent in keeping up with international standards, that determine data accuracy, thus allowing replicability and comparison. To make things slightly challenging, the competition for shiptime is intense since there are not many research vessels available. Thus, international cooperation is essential to access and deploy ocean technologies. Governments need to support researchers in negotiating equitable and fair platforms for sharing research infrastructure and co-developing marine technologies.

### The Legal Framework That Supports the Transfer of Marine Technology

There is a compelling international legal framework that aims at fostering the transfer of marine technologies, in particular in the context of the United Nations Law of the Sea Convention (LOSC). The LOSC provides a comprehensive framework regulating the jurisdiction of States Parties and activities taking place at sea, interacting with other instruments, actors and regimes (Trevisanut et al., 2020). Even though scientific evidence is interwoven in many provisions of the Convention, the transfer of marine science and technology is enshrined in part XIII (Marine scientific research), part XIV (Development and transfer of marine technology), and articles 143, and 144. Whereas the link between the framework on marine scientific research, transfer of technology and capacity development has been analyzed elsewhere (Harden-Davies and Snelgrove, 2020), the literature lacks a closer look into the special rules directed to developing countries.

The obligation of transferring marine technology generally covers 1) access to data, information and knowledge; 2) training human resources on science and technology; 3) promoting access to equipment and infrastructure; and 4) promoting international, regional and national scientific and technical cooperation (Harden-Davies and Snelgrove, 2020). In more details, within the framework of scientific cooperation, there is a special obligation for States, alone or in collaboration, to promote the flow of scientific data and information, as well as the transfer of knowledge resulting from MSR and transfer of marine science and technology to developing countries. Additionally, international efforts must focus on increasing the autonomous scientific capability and infrastructure of these countries through capacity development actions as well as the establishment of national and regional research centers aiming at not only increasing skills in pure science, but also to improve the social and economic development of these countries (art. 244 (2), art. 266 (1)(2), art. 268 (d), art. 275, art, 276 LOSC). Aligned with States, International Organizations must endeavor to conclude focused programmes of technical cooperation for transferring all kinds of marine technologies and technical assistance to States that have not been able to establish or promote their own technological capacities in pure or applied marine sciences (art. 269 (a)). Even when not intermediated by international organizations, the TMT between States must consider the needs and interests of developing countries (art. 272, LOSC). Article 267 provides means of interaction with other legal regimes by counterbalancing the obligation to transfer marine technology with the obligation of due regard the rights and duties of holders, suppliers and recipients of marine technology. Table 1 summarizes the provisions in parts XIII and XIV with rights and obligations for developing countries.

**TABLE 1:** Law of the Sea Convention provisions in part XIII and part XIV (Development and transfer of marine technology) specifically dealing with developing countries.

Special rules for developing States in part XIII	Art 244.2	States and IO shall transfer scientific data, information and knowledge States and IO shall strengthen the autonomous MSR capabilities of developing countries States and IO shall strengthen human resources of developing countries through education and training
Special rules for developing States in part XIV	Art 266	States shall promote the development of MS and technological capacity of States with regards to exploration, conservation and management
	Art 268	States, IO, ISA shall promote the development of HR through training and education
	Art 269	States, IO, ISA shall endeavour: establish progammes of technical cooperation - own technological capacity
	Art 272	IO shall coordinate Global or regional programmes taking into account interests and needs
	Art 273	States, OI and ISA shall facilitate the transfer of Skills and marine technology with regards to activities in the Area
	Art 275.1	States, IO, ISA shall establish national marine scientific and technologic research centres
	Art 276	States, IO and ISA shall promote the Establishment of regional marine scientific and technological research centres to stimulate and advance the conduct of MSR and foster the TMT

HR, Human Resources; IO, Intergovernmental Organizations; ISA, International Seabed Authority; TMT, Transfer of Marine Technology; MSR, Marine Scientific Research; MS, Marine Science.

Understanding that technological and scientific developments would require normative adaptation over time, article 271 calls for collaboration though international organizations for enacting criteria and guidelines to facilitate the TMT taking into account the interests and needs of developing countries, including skills and technology regarding activities in the Area, i.e., the seabed and ocean floor and subsoil thereof, beyond the limits of national jurisdiction. Even though no specific organization is mentioned in LOSC, IOC-UNESCO has acted as the focal point for implementing parts XIII and XIV. Other organizations with competences related to ocean sciences are the Food and Agriculture Organization (FAO), the International Seabed Authority (ISA) and the International Maritime Organization (IMO), among others with a more regional focus (Nordquist et al., 1985, pp. 558–560; United Nations, 2010). The conduct of MSR has increasingly been undertaken by cooperative arrangements, what is fostered by articles 424 and 244 of the Convention. Besides, IOC has been leading initiatives of capacity building in marine scientific research and has assumed a pivotal role in discussions in the BBNJ negotiations, which has transfer of technology and capacity building in the core of the negotiations (Harden-Davies, 2016).

In 1994, a new Implementing Agreement under LOSC was negotiated to implement Part XI regarding activities in the Area (United Nations, 1994). Developed countries were dissatisfied with the regime negotiated in LOSC for the Area, including the obligation of mandatory technology transfer. As part of the compromise to acquire the necessary number of ratifications for the LOSC to come into force, the 1994 Agreement modified article 144 introducing new principles in disfavor of developing countries (Galindo, 2006). First, it has linked the conditions to facilitate the access of technology to the terms of the open market or through joint-ventures, reducing favorable prices to developing countries. Second, it has submitted technology acquisition to the effective protection of property rights, one important limitation for TMT in current times, as we shall discuss below (United Nations, 1994). Despite the setbacks introduced by the 1994 Agreement, the ISA has established an Endowment Fund in 2006 to support the participation of scientists from developing countries in research projects (United Nations, 2010), which, in turn, has been subject to some criticism (Jaeckel et al., 2016).

In spite of the comprehensive legal framework favoring scientific cooperation and marine technology transfer with particular provisions focusing on increasing capacities in developing countries, part XIII and part XIV of the LOSC are under-implemented (Long, 2007) As a result, there is currently a lack of balance between developed and developing countries in producing ocean science (IOC-UNESCO, 2017). These concerns are vivid in many international stages, such as in the BBNJ negotiations, where countries of the Global South are requesting more legal opportunities for accessing marine technologies. As the scope of the Decade is broader than the BBNJ, we claim that it could act more ambitiously as a springboard to foster the implementation of the special rules on marine scientific research and transfer of technology for developing countries, particularly considering the rules on international scientific cooperation aforementioned and the positive outcomes to promote transfer of technology of informal arrangements.

### Challenges and Opportunities in Implementing the Transfer of Marine Technology

#### Implementing the LOSC Rules on Transfer of Marine Technology

Technology transfer can mean a diversity of processes. For example, it can be applied to a dual use of a certain technology being transferred from one field of application to another. It can also represent the factual physical movement of an asset (or even immaterial elements, such as know-how or technical information) or people or a set of capacities between places. Here, we will address technology transfer as the transfer of systematic knowledge for the manufacture of a product, for the application of a process or for the rendering of a service and does not extend to the mere sale or lease of goods (United Nations Conference on Trade and Development, 2014).

**Figure:**
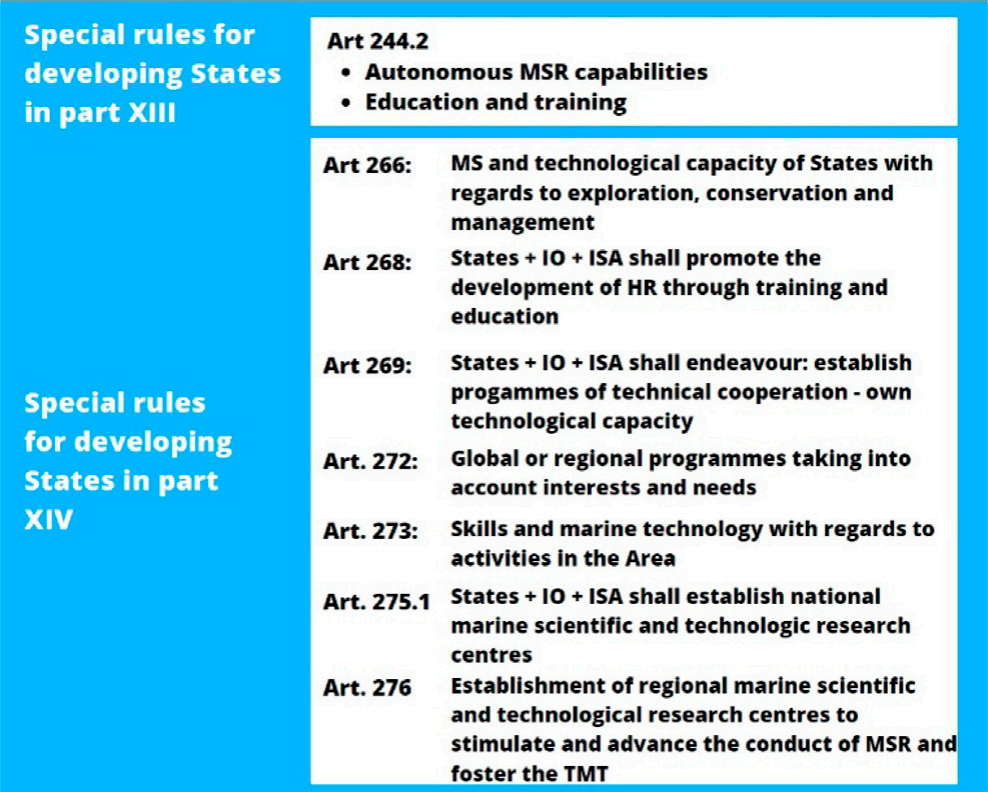


Marine technology transfer is generally referred to in the context of the IOC Criteria and Guidelines on the Transfer of Marine Technology, or GTMT, as illustrated in Box 1 (IOC-UNESCO, 2005). GTMT details the need for a clearing-house mechanism, by which interested stakeholders could identify technology-holders and technology needs among the global ocean community. This clearing-house mechanism is not yet established, although IOC has created a Group of Experts on Capacity Development that have produced recommendations on ways to move forward, based in other organizations’ models (IOC-UNESCO, 2019). IOC has, however, established a proof-of-concept trial clearing house mechanism in its regional body for the Latin America and the Caribbean through a dedicated website.^3^ This trial version makes available information on some of the region’s institutions, experts and research vessels, but a match making feature for those seeking available marine technologies from the North is inexistent. Therefore, after 15 years of the establishment of those criteria and guidelines, the world has yet to see transformational technology transfers that result in a balance between countries in the access and use of marine technologies (IOC-UNESCO, 2017; Salpin et al., 2018).

Diplomacy cannot afford to postpone the debate on the effective transfer of marine technologies. As the world’s population grows, there will be a race to explore the ocean natural resources further. Thus, ocean sustainable development based on the best available scientific knowledge is of utmost importance for future generations, in particular for developing countries (Hassanali, 2020). Bearing this in mind, the United Nations proclaimed the next decade as the UN Decade of Ocean Science for Sustainable Development (2021–2030).

The Decade of Ocean Science shall be a good opportunity to foster the debate around effective manners to progress in granting opportunities for developing countries to access marine technology and capacity development (Claudet et al., 2019), by implementing the regimes enshrined in part XIII and XIV of the LOSC. For this to happen, the implementation of the Decade should be centered in searching for equality in the access and use of marine technologies for sustainable development and human and environmental wellbeing. Terms such as co-development of technology instead of transfer, with a more equitable and linear participation of stakeholders, should also be promoted. In this sense, science diplomacy can inform on practices applicable to fostering this balance.

### Scientists Leading the Transfer of Marine Technology

In practice, marine technology transfer has relied less in formal intergovernmental diplomatic routes and more in peer-to-peer exchange. Peer-to-peer cooperation is a basic mechanism of the scientific endeavor. It has produced advancements in our common knowledge of the marine realm allowing society to make better informed decisions (Fischhoff and Scheufele, 2013). Research centers, universities and individual researchers have fostered technology transfer for problem-solving, aiming at progressing in scientific discovery. Agreements signed between research institutions and universities often include the exchange of human capacities and technology transfer at some level (Dolan, 2012). Drivers of such agreements are opportunities presented by the growing internationalization mechanisms adopted by those institutions (Qiang, 2003). Such mechanisms aim at projecting national capacities and competencies abroad to attract human and financial capital for further institutional developments, as a form of investment. In the context of Latin America and the Caribbean, internationalization has also provided the means to access foreign research funding and assets, placing an important opportunity to foster partnerships, but also to overcome national budget constraints.

**Figure:**
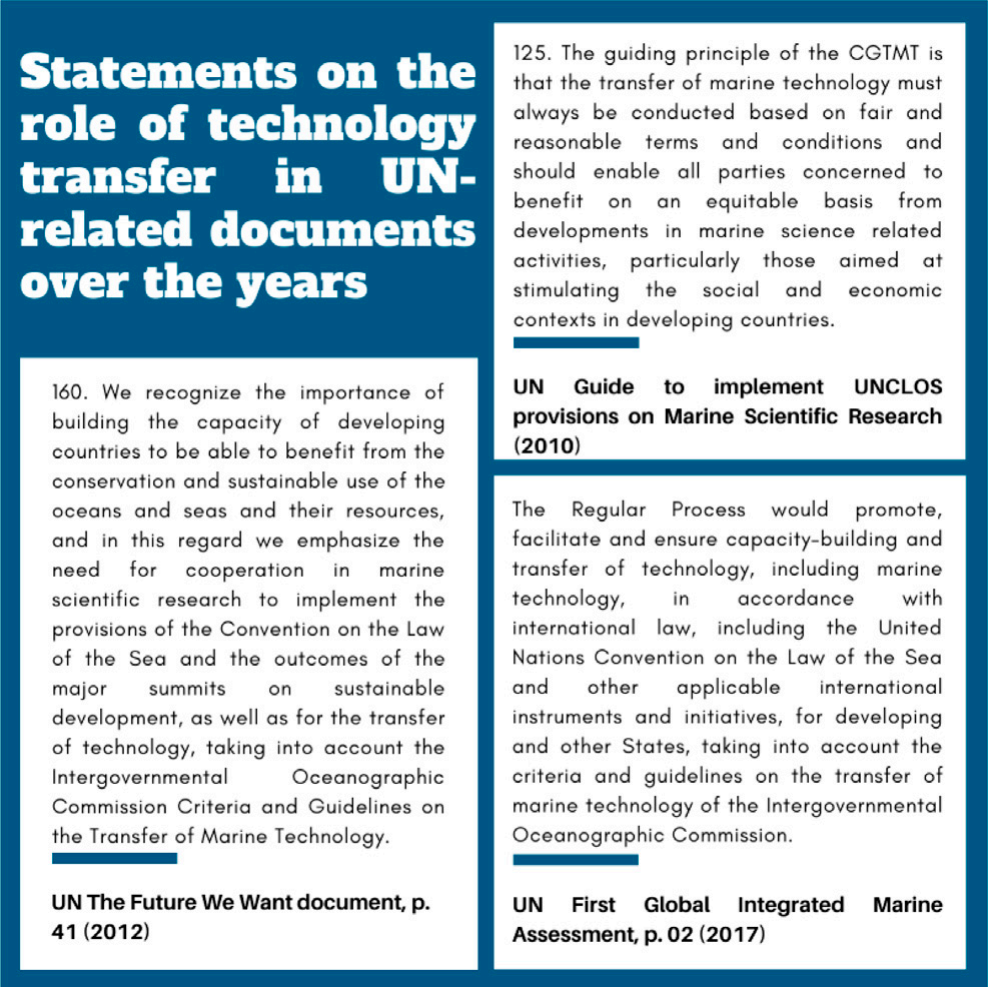


This practice is more common in the context of technologies developed by publicly funded research, mainly targeting scientific discovery. Privately funded research assets, in particular those aimed at exploring the marine resources such as oil, fisheries and minerals, are less common on those agreements because these technologies raise industry’s competitiveness and profit (Ruffini, 2020a). There are, however, a few privately funded organizations that use advanced technologies to promote open access information to society [e.g., Global Fishing Watch (Nugent, 2019)].

It is therefore fundamental that scientific cooperation in informal pathways is continued and promoted so science can profit from the free thinking and foster technology transfer. In fact, diplomacy should acknowledge and promote these informal channels where applicable, supporting actions that have been successful over time, such as cooperation agreements between research institutions. This informality is addressed as a form of Track 2 diplomacy in International Relations scholarship. The term can be understood as a parastatal informal diplomacy in which stakeholders are not necessarily bound to Governments (Jones, 2015). Track 2 diplomacy can use the science international cooperation to progress on addressing community and common interests in a more flexible way than the official, Government-led track 1 diplomacy. At the end of the day, both forms of negotiations should be interlinked and supportive of one another if we are to see change in the transfer of marine technologies during the Decade of Ocean Science, for example.

### Intellectual Property Rights (IPR)

The overarching difficulty for an intergovernmental body such as the IOC to pragmatically propose the transfer of marine technologies lays partially on issues of Intellectual Property Rights (IPR) (Zhou, 2019). Unlike the provisions on TMT, MSR and capacity development, under the scope of the LOSC and the mandate of institutions connected with this regime, IPR in under the mandate of the World Intellectual Property Organization (WIPO) and the World Trade Organization (WTO), through the Agreement on Trade-Related Aspects of Intellectual Property Rights (TRIPS). Indeed, as the LOSC is not a stand-alone treaty, it interacts with other regimes of international law, and has mechanisms to do so (Trevisanut et al., 2020), as for instance the above-mentioned article 267. Nonetheless, the conversation between these regimes has so far only favored private companies detaining patents.

In light of global environmental conundrums, WIPO was challenged to balance “the free transfer of technologies and sustainable innovation”, but without much success (Zhou, 2019). Similar process is undergoing in the WTO, and negotiations on technology transfer under the scope of TRIPS have not been evolving (Zhou, 2019). Therefore, traditional diplomacy has been unable to reach consensus on how to balance IPRs and public interests to advance sustainability (Latif et al., 2011).

### Private Sector Involvement

Companies take risks and make investments to profit from technological assets. The private sector alone should not be accountable to make change by opening patents and handling technology blueprints. In addition, countries in Latin America and the Caribbean will benefit little from blueprints if they do not possess the necessary human capacities and physical facilities to develop marine technologies. Therefore, an intergovernmental coordinated effort needs to be developed by finally operationalizing the clearing-house mechanism of IOC to then match technology holders and needs (Harden-Davies, 2016). Second, public diplomacy needs to foster a discussion on the possible trade-offs for the private sector to join in this effort. Companies can profit from opening new markets and investing in capacitating new labor in the region. Third, local governments need to invest in innovation policies and start-up programs to absorb the technology being transferred. Local business might then flourish, and local realities will adapt technologies to their needs, feedbacking the innovation process at a larger scale. At the end of this complex process, countries can begin to negotiate the co-development of technologies, beyond the scope of transferring technology as a passive-active relationship (Chesbrough and Schwartz, 2007). Although there are conflicting views addressing market competition and sustainability, there are also opportunities to leverage this relationship, such as private research programs on marine ecosystem restoration or pollution (Virdin et al., 2021).

Private companies’ interests are considered by diplomacy when defending national positions in international negotiations. Same applies to public interest, as the societal benefit of a healthy and safe ocean environment. Thus, diplomacy needs to balance community/public interest with those interests coming from specific groups or countries. This complex relationship between national interests and global public goods involving science and technology is taken under the scrutiny of science diplomacy research (Ruffini, 2020b). Moreover, a better coordination between international regimes such as LOSC, WIPO, and TRIPS is highly desired. The Decade of Ocean Science should open this dialogue by confronting diplomatic negotiations in both regimes and searching for opportunities. A simple recommendation in this issue would be to align country’s representations in both process with the aim of finding common grounds for opening this frank debate on Intellectual Property Rights.

## Discussion

The United Nations Law of the Sea Convention and related implementing instruments have set rights and obligations able to reduce worldwide asymmetries in the access to scientific knowledge and marine technology. Nevertheless, in spite of some increase in the participation of Asian countries in scientific publications, mentioned in the latest Global Ocean Science Report, the scientific and technological capabilities remain inequality distributed. Developed countries still concentrate the majority of ocean science human capacity and more incentives for researchers, like the access to international forums and networking (IOC-UNESCO, 2020a). Equally, only five countries in the world, all located in the global north, have full wide range access of technological infrastructure, with only a few others with capacity to conduct open waters and deep-sea research (IOC-UNESCO, 2020a). For instance, none of the Small Island Developing States (SIDS), which includes the Caribbean States, have deep-research vessels.

The origins of many of these difficulties in promoting the right of access to scientific knowledge and technology to developing countries lye in historical processes of colonization (Headrick, 1981). Additionally, from an epistemological perspective, science is a western invention, as so, from the starting point developing countries need to follow theories and methods founded in an alien mindset, still being under dispute how to integrate traditional and indigenous knowledge in the science-making (Weiss, 2005; Mulalap et al., 2020). This topic assesses whether science diplomacy is an appropriate tool to reduce scientific and technological asymmetries without disregarding the compelling reasons for a deeper discussion.

### Science Diplomacy Facilitating the Transfer of Marine Technologies in Latin America and the Caribbean

Latin America has experienced a raise in social sciences’ research in understanding the role of Science in advising policy, with a prominent focus on “center-periphery” relations in scientific research and the globalization of the social sciences, or the ownership of knowledge, particularly indigenous knowledge, when compared to the United States and Europe (Echeverria et al., 2020). Historically the theoretical field of International Relations (IR) has dealt with technology in both an optimistic and a skeptical conflict, in particular scholarship around the role of technology in the Cold War. Science and Technology was placed exogenously in theoretical IR and the dynamics and global impacts of Science needed further empirical evidence. Today, IR is seeking ways to incorporate the global politics of science and technology as a distinct subfield, which is by default an interdisciplinary approach that needs to include other fields of social sciences therein (Koh and Jayakumar, 1977). Therefore, science diplomacy can offer a new interdisciplinary approach to study how science and technology, its multiple facets and understandings, can influence international relations (Lidskog, 2014). We frame this discussion around the taxonomy provided by (The Royal Society, 2010) so the organization reflects the general science diplomacy literature.

First, “Diplomacy for science”, which stands for diplomacy facilitating international scientific cooperation by leveraging investment and prioritizing research to address uncertainties in decision-making. Here, diplomacy can set official frameworks by which countries can access marine technologies, such as through the IOC. By doing so, diplomatic negotiations can foster the establishment of international cooperation on fair and equitable grounds, in accordance with the Law of the Sea Convention. Moreover, diplomacy needs to integrate debates going on in different fora, in particular among WTO and WIPO, on how to deal with intellectual property rights. In addition, diplomacy can foster an arrangement between the public and private sector regarding the access and application of relevant technology to research global public goods, such as the ocean. Ocean science can only progress in an equitable manner if access to marine technologies is granted on an equitable basis through the diplomatic decision making. Thus, diplomacy for science in this scenario means intergovernmental negotiations to grant access to marine technologies and capacity development.

Second, “science in diplomacy”, that deals with the provision of scientific evidence to support international decision-making. Research will be responsible to inform diplomacy on the above mentioned negotiations. Knowledge gaps and trending themes of concern need to be communicated in such a way that diplomacy can discuss institutional and legal arrangements to overcome current obstacles for an effective transfer of marine technologies. Scientists have a pivotal role in clarifying what should be the results in effective marine technology transfer, highlighting the current pathways to acquire technologies and barriers, such as Intellectual Property, maintenance and operating costs. Non-governmental organizations and intergovernmental organizations shall play an important role in this regard (Lidskog, 2014). For example, the organization of public debates among scientists using the networks under NGOs are theme-oriented and independent from States and formal diplomacy, resulting in a flexible approach to discussing the state-of-the-art research and potential future actions. In ocean affairs, NGOs have provided scientific expertize since the early negotiations of the LOSC (Koh and Jayakumar, 1977). Therefore, science in diplomacy will allow provision of knowledge gaps and current technology needs to properly advance in ocean sustainability to comply with global community interests.

Lastly, “science for diplomacy”, in which international collaboration advances to bridge countries and build a constructive dialogue through joint research projects. The utmost example of such is the adoption of the UN Decade of Ocean Science. The Decade is hoped to be the long-waited opportunity for research to bridge countries and people around a common goal. Different stakeholders with diverse values and needs shall inform the Decade’s process on achieving societal goals of ocean sustainability (Claudet et al., 2019). The Decade’s *raison d’être* is to put ocean science in service of society, including policy making, despite any possible tension between countries in other international debates. Thus, science for diplomacy will act to allow this dialogue between countries and stakeholders to take place through joint regional/global research efforts, that can be fostered initially by informal pathways, attained to the Track 2 diplomacy practices.

Ultimately, the balance between national political interests and global community interests in transferring marine technologies to foster ocean sustainability is a matter of balancing competition versus cooperation (Ruffini 2020b). There must be an optimal point in which trade-offs are made and commitments are adopted. This point must be achieved by addressing both the issues of national priorities, such as industry development and labor enhancement, with those of global concern, such as marine environmental protection and ecosystem service restoration. In this regard, scientists become yet another social group with intrinsic values and interests (Jasanoff, 1987; McCain, 2016, pp. 253‐257). Therefore, progressing in understanding the social dynamics within the group of scientists and between scientists and diplomatic relations becomes essential to better inform global processes based on scientific evidence, such as the UN Decade of Ocean Science (Rose, 2018). Science diplomacy research in this regard, and in particular in the context of Latin America and the Caribbean, the region’s gaps and priorities, will enhance the global discussion to implement the Decade.

### Examples of Science Diplomacy Processes Leading the Transfer of Marine Technology

Peer-to-peer cooperation agreements between research institutions and universities generally include the exchange of human capacities and technology transfer at some level (Dolan, 2012). Drivers of such agreements are opportunities presented by the growing internationalization mechanisms adopted by those institutions (Qiang, 2003). Internationalization of universities and research centers is one of the outcomes of the globalization of science.

A good example of such is the cooperation between research institutions from Germany and Cape Verde to create and operate an ocean research center in Cape Verde (Kaehlert et al., 2017). The Ocean Science Center Mindelo results from a formal agreement between the GEOMAR Helmholtz Center for Ocean Research and Cape Verde’s Instituto do Mar—IMar. The Tropical portion of the Atlantic has a determinant role in the heat exchange between the ocean and the atmosphere, a feature that is central to understand global climate and ocean dynamics (Seidel et al., 2008). German scientists wish to access an island in the middle of the Atlantic to further enlighten how the Tropical Atlantic influences the North. Germany benefits from relevant information and Cape Verde with the access to technologies and capacities to deal with their own waters. Moreover, the center is devoted on building capacities in Cape Verde so their ocean science community can be empowered. Ultimately, the German interest in Cape Verde contributed to the European Commission signing a diplomatic bilateral science and technology agreement on ocean research as a part of a broader ocean science diplomacy arrangement for the whole Atlantic basin (Polejack et al., 2021). This ocean science diplomacy practice has balanced the capacity needs of Cape Verde with the German interests in the region advancing knowledge production that will be fit for the global ocean assessment purpose, fully implementing articles 244, 266 and 275, LOSC.

Another good example of science diplomacy aiding countries to implement their international obligations in the transfer of marine technologies is the global ocean observation network. Ocean observations are highly dependent on technology and, under the auspices of IOC’s Global Ocean Observing System (GOOS) cooperation has been key to deploy equipment worldwide, such as buoys, drifters and other ocean monitoring instruments (Tanhua et al., 2019). In general, this cooperation involves the exchange, maintenance and calibration of equipment from one country to another. The handling of equipment’s blueprints for local development and manufacture is much rarer. Among the practical examples of our knowledge is the development of the Atlas-B buoy in Brazil (Campos et al., 2014). The U.S. National Oceanic and Atmospheric Administration (NOAA) freely handed the blueprints of their Atlas buoy technology for development in Brazil. As a result, Academia and industry partnered to develop an adaptation of this equipment, which was deployed in face of Brazil for testing. In spite of formal Government agreements in this matter, both NOAA and the University of São Paulo together with two Brazilian companies were able to successfully transfer a key technology nonexistent in the country before. Capacities were developed and today Brazil is able to progress in the manufacture of this buoy.

From the above mentioned, science diplomacy as a practice provides different perspectives of implementing the international obligations of transferring marine scientific knowledge and technology, reducing inequalities and empowering developing countries. Practical examples support this perspective, although the Decade will be a more ambitious stage for the science diplomacy interplay.

## Conclusion

Marine researchers in Latin America and the Caribbean struggle to conduct state-of-the-art research mostly due to the lack of permanent funding, appropriate scientific capacities and access to marine technologies. Consequently, these countries are challenged to contribute with scientific evidence in current ocean affairs, such as the BBNJ negotiations (Harden-Davies and Snelgrove, 2020). Although the global ocean governance framework provides the legal and institutional support for the transfer of marine technology from developed to developing countries aiming at strengthening local and regional capabilities, after decades of the entry into force of LOSC, part XIII and part XIV are considered among the least implemented of the LOSC (Long, 2007; Long and Chaves, 2015).

The globalized research community has provided informal venues for the transfer of marine technology. However, these peer-to-peer relationships will not be sufficient to achieve the equity that several States have called for to strength national capacity permanently to meet national needs and international standards. Therefore, this paper presents some concrete recommendations on how countries in Latin America and the Caribbean can enhance their national scientific capacities by using science diplomacy as a tool to foster beneficial international deals.

First, according to the requirements of the LOSC and the Resolution on the development of national marine science, technology and ocean service infrastructure (A/CONF.62/120*), developing countries must produce science and technology needs assessments, by which gaps and priorities shall be apparent. Such an effort could be supported by international organizations, the scientific community and research organizations, including from the private sector, together with governments.

Second, efforts must be taken to effectively implement the clearing house mechanism as per the IOC guidelines (IOC-UNESCO, 2005). Major technology holders from the developed world and representatives from organizations with mandate related to intellectual property, such as WTO and WIPO, should be included in discussions on the of such a clearing house mechanism, providing inputs and other perspectives. Issues related to exchange rate, taxation, fees for transportation, and limits to comply with standards for ocean observation should be considered in the clearing house mechanism. Additionally, it is relevant to discuss about incentives to create regional certified laboratories in developing countries to provide maintenance and calibration for equipment, as well as reviewing the standards for accreditation. Latin America and the Caribbean can profit from the trial version of this mechanisms that IOC has initialized in the region.

Third, a shift in vocabulary may represent a positive change on how developed countries understand their role in promoting scientific and technological equity. Using terminologies such as co-development of technology instead of transfer are able to build more linear relations between stakeholders and reduce perspectives of subservience (center-periphery).

The Decade of Ocean Science shall be a good opportunity to foster the debate around effective manners to progress in granting opportunities for developing countries to access marine technology and capacity development, by implementing the regimes enshrined in part XIII and XIV of the LOSC. Countries in Latin America and the Caribbean have the opportunity during this Decade to push for improvements in the access of marine technologies. The provisions in the LOSC and related instruments give the legal basis for this discussion. Moreover, ocean science diplomacy can provide the necessary insights on possible negotiations based on evidence and favoring fair and just transition pathways.

## Footnotes

1 For the purpose of this paper, marine technology encompasses the “instruments, equipment, vessels, processes and methodologies required to produce and use knowledge to improve the study and understanding of the nature and resources of the ocean and coastal areas” (IOC-UNESCO, 2005, p. 9)

2 In the absence of a clear-cut definition of marine scientific research in the United Nations Law of the Sea Convention (LOSC), we understand this activity as “any study or related experimental work designed to increase [hu]man’s knowledge of the marine environment” (Soons, 1982)

3
http://portete.invemar.org.co/chm, accessed on January 27, 2021.
