# Corrigendum: Ocean Science Diplomacy can Be a Game Changer to Promote the Access to Marine Technology in Latin America and the Caribbean

**DOI:** 10.3389/frma.2021.711473

**Published:** 2021-07-13

**Authors:** Andrei Polejack, Luciana Fernandes Coelho

**Affiliations:** ^1^ WMU-Sasakawa Global Ocean Institute, World Maritime University, Malmö, Sweden; ^2^ Ministério da Ciência, Tecnologia e Inovações, Brasília, Brazil; ^3^ Research Group Natural Resources, Law, and Sustainable Development, Brazilian Institute for the Law of the Sea, Salvador, Brazil

**Keywords:** science diplomacy, access to technology, Latin America, Caribbean, UN decade of ocean science

In the published article, there was an error. A correction has been made to the **Affiliation 3**.

Instead of “Research Group Natural Resources, Law, and Sustainable Development, Brazilian Institute for the Law of the Sea, *Caxias do Sul*, Brazil,” it should be “*Research Group Natural Resources, Law, and Sustainable Development, Brazilian Institute for the Law of the Sea, Salvador, Brazil*.”

The authors apologize for this error and state that this does not change the scientific conclusions of the article in any way. The original article has been updated.

